# Endospores associated with deep seabed geofluid features in the eastern Gulf of Mexico

**DOI:** 10.1111/gbi.12517

**Published:** 2022-08-22

**Authors:** Jayne E. Rattray, Anirban Chakraborty, Gretta Elizondo, Emily Ellefson, Bernie Bernard, James Brooks, Casey R. J. Hubert

**Affiliations:** ^1^ Department of Biological Sciences University of Calgary Calgary Alberta Canada; ^2^ TDI Brooks International College Station Texas USA; ^3^ Department of Biological Sciences Idaho State University Pocatello Idaho USA; ^4^ Geological Sciences Stanford University Stanford California USA

## Abstract

Recent studies have reported up to 1.9 × 10^29^ bacterial endospores in the upper kilometre of deep subseafloor marine sediments, however, little is understood about their origin and dispersal. In cold ocean environments, the presence of thermospores (endospores produced by thermophilic bacteria) suggests that distribution is governed by passive migration from warm anoxic sources possibly facilitated by geofluid flow, such as advective hydrocarbon seepage sourced from petroleum deposits deeper in the subsurface. This study assesses this hypothesis by measuring endospore abundance and distribution across 60 sites in Eastern Gulf of Mexico (EGM) sediments using a combination of the endospore biomarker 2,6‐pyridine dicarboxylic acid or ‘dipicolinic acid’ (DPA), sequencing 16S rRNA genes of thermospores germinated in 50°C sediment incubations, petroleum geochemistry in the sediments and acoustic seabed data from sub‐bottom profiling. High endospore abundance is associated with geologically active conduit features (mud volcanoes, pockmarks, escarpments and fault systems), consistent with subsurface fluid flow dispersing endospores from deep warm sources up into the cold ocean. Thermospores identified at conduit sites were most closely related to bacteria associated with the deep biosphere habitats including hydrocarbon systems. The high endospore abundance at geological seep features demonstrated here suggests that recalcitrant endospores and their chemical components (such as DPA) can be used in concert with geochemical and geophysical analyses to locate discharging seafloor features. This multiproxy approach can be used to better understand patterns of advective fluid flow in regions with complex geology like the EGM basin.

## 1 INTRODUCTION

The ability to transform from active cells into endospores (resistant semi‐dormant non‐reproductive structures) allows some bacteria to survive exposure to extreme environmental conditions, while retaining the ability to re‐activate to fully metabolising cells when conditions are favourable. Quantification of in situ endospore populations in sediments and soils can be achieved by measuring concentrations of the endospore‐specific biomarker 2,6‐pyridine dicarboxylic acid or dipicolinic acid (DPA; Fichtel, Koster, Rullkotter, et al., 2007; Fichtel, Koster, Scholz‐Bottcher, et al., 2007; Heuer et al., 2020; Lomstein & Jørgensen, 2012; Lomstein et al., 2012; Rattray et al., 2021; Volpi et al., 2017; Wörmer et al., 2019; Yang & Ponce, 2011). DPA constitutes up to 20% of endospore core dry weight and functions as a biological packing material to prevent endospore lysis during dormancy (Setlow et al., 2006; Setlow, 2007, 2011). Recently, in global deep marine sediments, DPA was used to estimate the presence of between 2.5 × 10^28^ and 1.9 × 10^29^ endospores in the uppermost kilometre of the global seabed. Endospore abundance increases with burial depth in marine sediments and is estimated to surpass numbers of non‐sporulated vegetative cells deeper than 25 m below seafloor (mbsf; Wörmer et al., 2019). Endospores have also been shown to be prevalent in more extreme environments, for example in 1.2 kmbsf and 120°C hot sediment layers in the Nankai Trough subduction zone (Heuer et al., 2020) where, in samples from depths warmer than 45°C, the abundance of vegetative cells dropped by two orders of magnitude (from 10^3^ cells/cm^3^ to ≤16 cells/cm^3^) and were dwarfed by endospore populations estimated at 1.2 × 10^6^ cm^−3^. Despite endospores being one of the most abundant life forms in the deep biosphere (Heuer et al., 2020; Lomstein et al., 2012; Wörmer et al., 2019), little is known about their biogeography and origins.

Gas and fluid expulsion from deep sediment layers have been proposed as processes facilitating the passive dispersal of microorganisms in EGM deep sea sediments (Chakraborty et al., 2018, 2020). Rapid changes in gas expulsion and potentially toxic hydrocarbons and metal species (due to mineral dissolution during upward fluid flow) suggest that organisms employing survival strategies will be well equipped to survive associated changes in their surroundings. Sporulating bacteria capable of metabolising in cold ocean bottom waters and the seabed (ca. ~2°C) are classified as psychrophilic or psychrotolerant (i.e. withstanding 0–20°C with optimum growth of psychrophiles around 5°C) and could thus be referred to as ‘psychrospores’ in their sporulated state when surroundings change to be less permissive for metabolic activity. Alternatively, sporulating thermophiles with the ability to metabolise at high temperatures may form ‘thermospores’ if subjected to less favourable growing conditions or upon dispersal away from warm environments. As small recalcitrant ‘propagules’ (endospores propagate bacteria to the next life cycle stage) undergoing passive dispersal prior to eventual resuscitation, endospores can potentially be transported for long distances throughout the ocean by global circulation patterns (Muller et al., 2014).

The EGM seabed system is geologically diverse, and endospores may have complex dispersal histories including migration from well‐known oil reservoirs in this basin, as well as other warm environments where they may have existed as vegetative cells. Bacteria belonging to the phylum *Firmicutes* inhabit a diverse range of environments ranging from the human gut (Forster et al., 2019; Mariat et al., 2009; Reichardt et al., 2014) to deep biosphere marine sediments (Heuer et al., 2020; Hoshino et al., 2020; Kapili et al., 2020; Wörmer et al., 2019). DPA analysis captures all endospores present in a sample regardless of environmental types and temperatures (e.g. wastewater treatment plants, riverine sediment and deep‐water marine sediments), that is regardless of whether they are psychrospores, mesospores or thermospores. In this study, endospore populations were quantified in marine sediments using the DPA proxy. Thermospore gene sequences (previously identified in the same sediments; Chakraborty et al., 2018) were re‐analysed at higher resolution to shed additional light on the dispersal history of specific endospore populations and assess potential source environments associated with geological features and subsurface fluid flow.

## 2 MATERIALS AND METHODS

### 2.1 Sample collection

**FIGURE 1:**
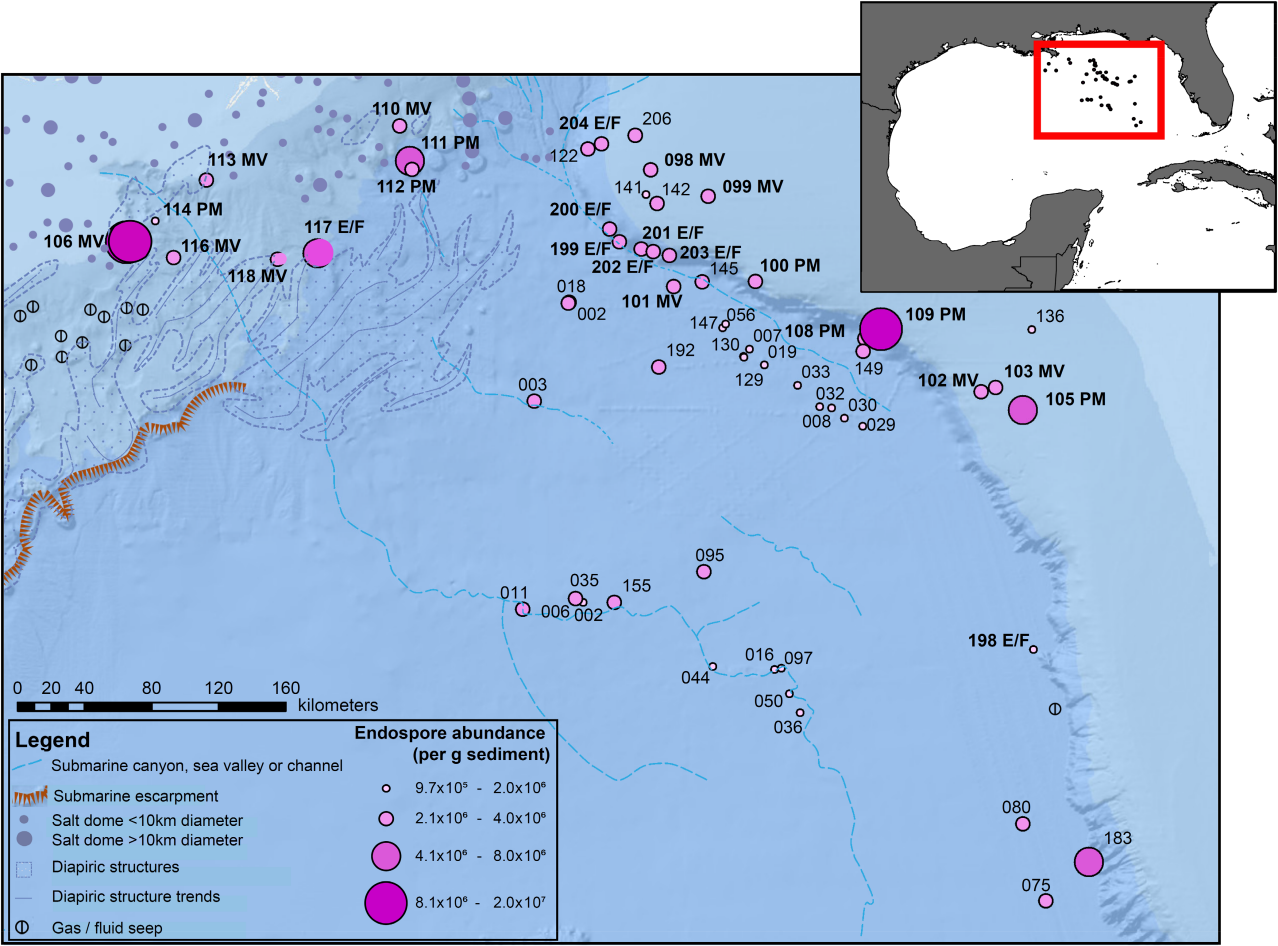
Endospore abundance per g sediment indicated by the size and shading of purple dots representing 60 sediment coring locations in the Eastern Gulf of Mexico. Each measurement represents a mean of triplicate dipicolinic acid measurements performed on separate sub‐samples of sediment from the same core. Locations identified as potential conduits for subsurface fluid flow are highlighted with bold station names as being associated mud volcano (MV), pockmark (PM) or escarpment/fault (E/F) features. Sampling locations are overlaid on a geologic map of North America (from Garrity & Soller, 2009) showing bathymetry and other seafloor features (e.g., salt domes and known gas/fluid seeps in the NW part of the map) as indicated in the legend. The inset map shows the sampling area (red box) relative to the entire Gulf of Mexico basin.

Sediment samples were selected from an offshore survey in the EGM conducted by the TDI‐Brooks International ‘Eastern Gulf of Mexico Consortium’ aboard the RV GeoExplorer in 2011 (Figure 1). Sediment from 60 different locations, ranging from 100 to 3300 m water depth and separated from each other by 6 to 600 km apart, were selected for this study.

### 2.2 Sub‐bottom feature analysis

**FIGURE 2:**
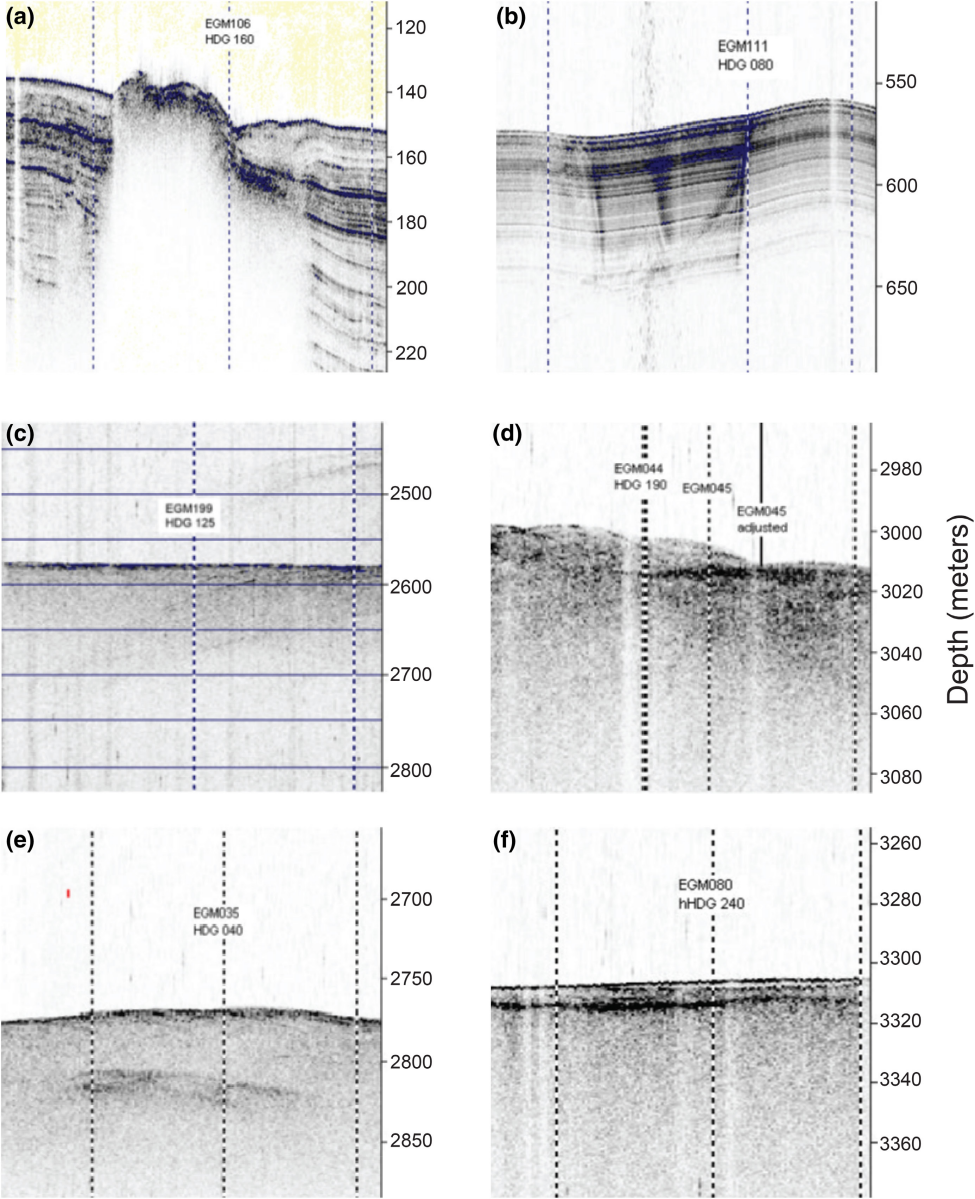
Examples of seabed geological structures determined using acoustic seabed data obtained from survey lines deployed with a compressed high intensity radiated pulse (CHIRP) sub‐bottom profiler. (a) Image from mud volcano site EGM106 showing raised sediment mound above the blank area of the mud volcano conduit. Vertical blue lines above the mound are caused by the flow of hydrocarbons and fluids ejected by the volcano into the bottom water. The sediment sample was dark grey with low water content and contained noticeable hydrogen sulphide. (b) Pockmark site EGM111 shows the vertical grey wavy lines caused by hydrocarbons flowing up to the seafloor. Dark areas of sediment indicate vertical “gas chimneys” within the depressed site. Sediment was mainly grey clay and contained foraminifera. Lower sections of the 5.4 m core contained traces of oil and observable hydrocarbon gas and hydrogen sulphide. (c) Escarpment/fault site EGM199 shows the flat seabed at the bottom of Florida escarpment cliff. The sediment was mainly dark green clay and contained foraminifera. Lower sections of this 5.4 m core contained traces of hydrogen sulphide. (d) Mound site EGM045 showing medium and high relief mounds with no visible conduits. (e) Subsurface seismic anomaly site EGM035 indicated by dark patches in the centre of the image. The sediment at this station was silty clay with sand and had moderate cohesion. (f) EGM080 is an example of a site with no discernible feature. Darker layers observed in the sediment subsurface can be caused by organic compounds depositing and accumulating or due to other differences in the sediment composition. Panels (a) and (b) are examples of conduit sites containing a direct vertical migration route for hydrocarbons to the seafloor (*n* = 25 out of the 60 sediments in this study), panels (c) to (f) display no visible conduits (*n* = 35 out of 60).

Acoustic seabed data were obtained by deploying survey lines with a compressed high intensity radiated pulse (CHIRP) sub‐bottom profiler. Ship speed during data acquisition was typically 5–7 knots. Data were interpreted together with seismic records to validate the site feature interpretation. Examples of sub‐bottom features observed using CHIRP analysis are shown in Figure 2.

### 2.3 DPA sampling and analysis

Sediment samples were collected by piston coring then sectioned, subsampled and frozen at −20°C, followed by freeze drying. Freeze dried sediment samples were subsampled in triplicate for DPA extraction using acid hydrolysis by combining 0.1 g sediment with 1000 μl 6 m hydrochloric acid and 1000 μl ultrapure water and then heating at 95°C for 4 h. Small volumes (400 μl) of hydrolysate were subjected to multiple rounds of additional freeze drying and then reconstituted in 1000 μl ultrapure water to remove any remaining acid. The dried extract was dissolved in 350 μl 1 m sodium acetate to which 40 μl of aluminium chloride (also in 1 m sodium acetate) was added to bind residual phosphate. The resulting solution was filtered through 0.2 μm Teflon syringe filters and 250 μl was used for analysis. Finally, 750 μl 10 mm Tb^3+^ in 1 m sodium acetate was added as a DPA chelation solution. DPA was analysed using a HPLC fluorescence Tb^3+^ chelation method (Rattray et al., 2021) using a Thermo UltiMate 3000 HPLC system coupled to a Thermo UltiMate 3000 FLD fluorescence detector with emission and excitation wavelengths of 270 and 545 nm, respectively. Separation of DPA and the Tb^3+^ chelate was achieved using a Phenomenex Kinetex® 2.6 μm EVO C18 column, 100 Å, 150 × 4.6 mm, attached to a guard column. This method can achieve low limits of DPA detection (~0.04 nm DPA) in 50 μl sample volume over a 10‐min analytical run time (Rattray et al., 2021). Peaks were integrated using chromeleon 7.0 software. Endospore numbers were calculated by assuming 2.24 femtomoles DPA per endospore, a conversation factor published by (Fichtel, Koster, Rullkotter, et al., 2007) and used in several other studies (e.g. Heuer et al., 2020; Wörmer et al., 2019) notwithstanding evidence for variability between endospores affiliated with different bacterial strains (Fichtel, Koster, Scholz‐Bottcher, et al., 2007; Rattray et al., 2021).

### 2.4 Geochemistry analysis

Sampling and analysis of geochemistry, anion and geographical data have been described in detail elsewhere (Chakraborty et al., 2018, 2020). In brief, piston cores of ca. 4.5–5.5 m depth were extruded in 20 cm sections. Surface sediments were sealed in sterile Whirl‐Pak bags and frozen at −20°C. Light hydrocarbon gases were analysed in the deepest core section by placing sediment in 500 ml gas canisters which were purged with nitrogen and stored at −20°C. Headspace gas and biomarker analysis was performed by Geomark Inc. Headspace gaseous alkanes were analysed directly from the sampling can using gas chromatography flame ionisation detection (GC/FID). Sediment hydrocarbons were initially analysed using total scanning fluorescence (TSF), and then liquid hydrocarbons were extracted using ACE solvent extraction and analysed using gas chromatography–mass spectrometry (GC/MS). Sediments were classified as oil positive based on a TSF maximum intensity threshold of 50,000 intensity units and a GC/MS unresolved complex mixture (UCM) threshold of 10 μg/g sediment. Sediments presenting TSF and UCM below these values were classed as oil negative for thermogenic hydrocarbons.

### 2.5 Sediment heavy metal extraction and analysis

Heavy metals, which are prevalent in crude oils, can be used as proxies to understand the origins and processes of oil formation (dos Anjos et al., 2018; Maryutina & Timerbaev, 2017; Mdluli et al., 2020; Walkner et al., 2017). In marine sediments, higher concentrations of heavy metals are found in deep‐to‐shallow conduits such as faults, trenches and subduction zones due to mineral and associated metal dissolution during upward fluid flow (Cangemi et al., 2010; Welty et al., 2018). In this study, heavy metals were assessed in 27 of the 60 sediment samples by sequential extraction using procedures of Benitez and Dubois (1999) and Zimmerman and Weindorf (2010). All reagents used for extraction were ultrapure trace metal standard grade and equipment was acid‐washed and rinsed in ultrapure water prior to extraction. Sample blanks were included to quantify trace metals originating from the extraction procedure and all data were blank‐corrected. For each sample, 1 g of sediment was extracted with 30 ml 0.1 m sodium nitrate, shaken and incubated at room temperature for 1.5 h. The sample was centrifuged for 10 min at ~1000 *g* (2800 rpm) and the supernatant decanted into an acid‐washed tube and the process repeated. Subsequently the organic fraction was extracted from the sediment with the same procedure but using 0.1 m sodium pyrophosphate tetrabasic as the extraction solvent. Extracts were diluted in HNO_3_, and metals and cations were analysed using a triple quadrupole ICP/MS, 8900 ICP‐QQQ (Agilent). Sediment metal concentrations were quantified using certified metal standards. Heavy metal concentrations were obtained for arsenic, cobalt, chromium, copper, gallium, nickel, molybdenum and zinc.

### 2.6 Sediment porewater chemistry, high‐temperature incubations and DNA sequencing

The procedure for the extraction and processing of sediment chemistry, enrichment culture samples and DNA extraction in the EGM sediment have been described in detail elsewhere (Chakraborty et al., 2018). Chloride and sulphate concentrations were analysed in filtered (0.2 μm) sediment porewater using ion chromatography with a Thermo Scientific Dionex ICS‐5000+ Ion Chromatography system.

Surface sediments used in microbiological analyses were stored in sterile Whirl‐Pak bags and frozen at −20°C until analysis. Slurries were prepared using 42 of the 60 sediment samples. Subsamples of these sediments were prepared as slurries in artificial seawater medium modified with 20 mm sulphate and six organic acids (acetate, butyrate, formate, lactate, propionate and succinate), each at a final concentration of 5 mm. Sediment slurries were pasteurised at 80°C in a water bath to select for endospores and then incubated at 50°C for 14 days. Genomic DNA was extracted before or after heating using the DNeasy PowerLyzer PowerSoil kit (Qiagen). Following PCR amplification of the v3‐4 region of the bacterial 16S rRNA gene using primers SD‐Bact‐341‐bS17 and SDBact‐785‐aA21 (Klindworth et al., 2012), amplicons were sequenced using an in‐house MiSeq benchtop sequencer (Illumina Inc.). Raw amplicon sequences are available through the NCBI Sequence Read Archive (Bio‐project accession number PRJNA415828).

### 2.7 Amplicon sequence variant (ASV) determination and statistical analysis

Paired‐end Illumina MiSeq reads corresponding to before and after heating of the 42 sediment samples were prepared by trimming and filtering demultiplexed fastq files. ASVs defined as amplified DNA sequences that are identical to each other (no mismatches) were then obtained from paired‐end raw reads using the Divisive Amplicon Denoising Algorithm 2 (DADA2) open‐source web‐based bioinformatics pipeline (Callahan et al., 2016). ASV data were processed and analysed using R package version 4.2.1 (RStudio Team, 2020). A Shapiro–Wilk test showed ASV sequences and endospore abundances were not normally distributed over the data sets, and all subsequent analysis was performed using non‐parametric statistical analyses, including Phyloseq (McMurdie & Holmes, 2013), ggplot2 (Wickham, 2016) and vegan (Oksanen et al., 2014).

Due to the compositional nature of high‐throughput sequencing data, the absolute abundance of DNA molecules originally existing in the environment cannot be determined using nucleic acid sequencing (Gloor et al., 2017). Numbers of sequence reads therefore represent the proportion of a given ASV within a sample. Differences in total counts observed or sample read depth can influence the proportion of ASVs per sample and lead to spurious associations. Errors introduced when using read depth can be mitigated by applying ‘rarefaction’ or read count subsampling to a common read depth (Lozupone et al., 2011; Wong et al., 2016). Disadvantages of rarefaction data set subsampling include substantial loss of information (McMurdie & Holmes, 2014) and removal of rare taxa. Rarefaction of the dataset obtained here would result in >60% of sequences being disregarded. Therefore, to identify novel, often rare, *Firmicutes* sequences in the heated sediment incubations, ASV counts were determined from the raw read data and plotted as centred log ratios (CLR; Gloor et al., 2017), which is a useful alternative to rarefaction (Aitchison, 1982). A caveat of CLR is that during log transformation information on the precision of the data is lost; however, the ratio remains the same irrespective of whether the data came from a large or small number of reads in a given sample library (Gloor et al., 2017). Data set read count zero values were replaced with the calculated median of the sample raw reads, which operates as a pseudo‐count prior to log transformation. Thus, instead of assigning an arbitrary value of one or zero counts, the pseudo‐count method is modelled to the variability in each sample (Kaul et al., 2017).

## 3 RESULTS

### 3.1 EGM seafloor geological features

This study's 60 sediment sampling locations (Figure 1) were classified into six seafloor feature types based on CHIRP and seismic data (Figure 2). The six types are mud volcanoes (*n* = 9; Figure 2a), pockmarks (*n* = 8; Figure 2b), escarpments/faults (*n* = 8; Figure 2c), mounds (*n* = 14; Figure 2d), subsurface seismic anomalies (*n* = 14; Figure 2e) and sediments with no feature (*n* = 7; Figure 2f). Mud volcanoes, pockmarks, escarpments and faults were additionally grouped together as conduit features, that is seabed geological structures that potentially offer a fluid migration pathway. Mud volcanoes are here distinguished from seafloor mounds by the presence of a seismic chimney (Figure 2a), as described by Niemann and Boetius (2010). Pockmarks are negative relief seafloor morphologies that form in association with sudden fluid expulsion events (Figure 2b; Hovland & Judd, 1988; King & Maclean, 1970). Submarine escarpments are usually formed by either faulting or erosion processes (Nash, 2013). Included in this study are the massive Florida escarpment (3200 m high cliff), as well as smaller escarpment structures across the EGM basin (Figure 2c). Submarine faults are scattered across the EGM seafloor with many fault systems associated with salt diapir formations (Shanmugam, 2021). Sites containing the seafloor structures shown in Figure 2a–c provide conduit fluid migration pathways for significant transportation of hydrocarbons and other fluids to the seafloor. Geological features in this study that have no direct association with conduits include mounds, subsurface seismic anomalies and sediments with no features (Figure 2d–f). Mounds are positive relief seafloor morphologies often associated with deep seismic chimneys, gas hydrate formations or mineral deposits, but lack a visible migration pathway (Figure 2d). Subsurface seismic anomalies are observations of high‐amplitude anomalous reflections identified using CHIRP that otherwise do not fall into one of the other categories above (Figure 2e). Sites with no features are areas of the seabed with no specific geological morphology or seismic anomalies (Figure 2f).

### 3.2 Endospore abundance in EGM surface sediments

**FIGURE 3:**
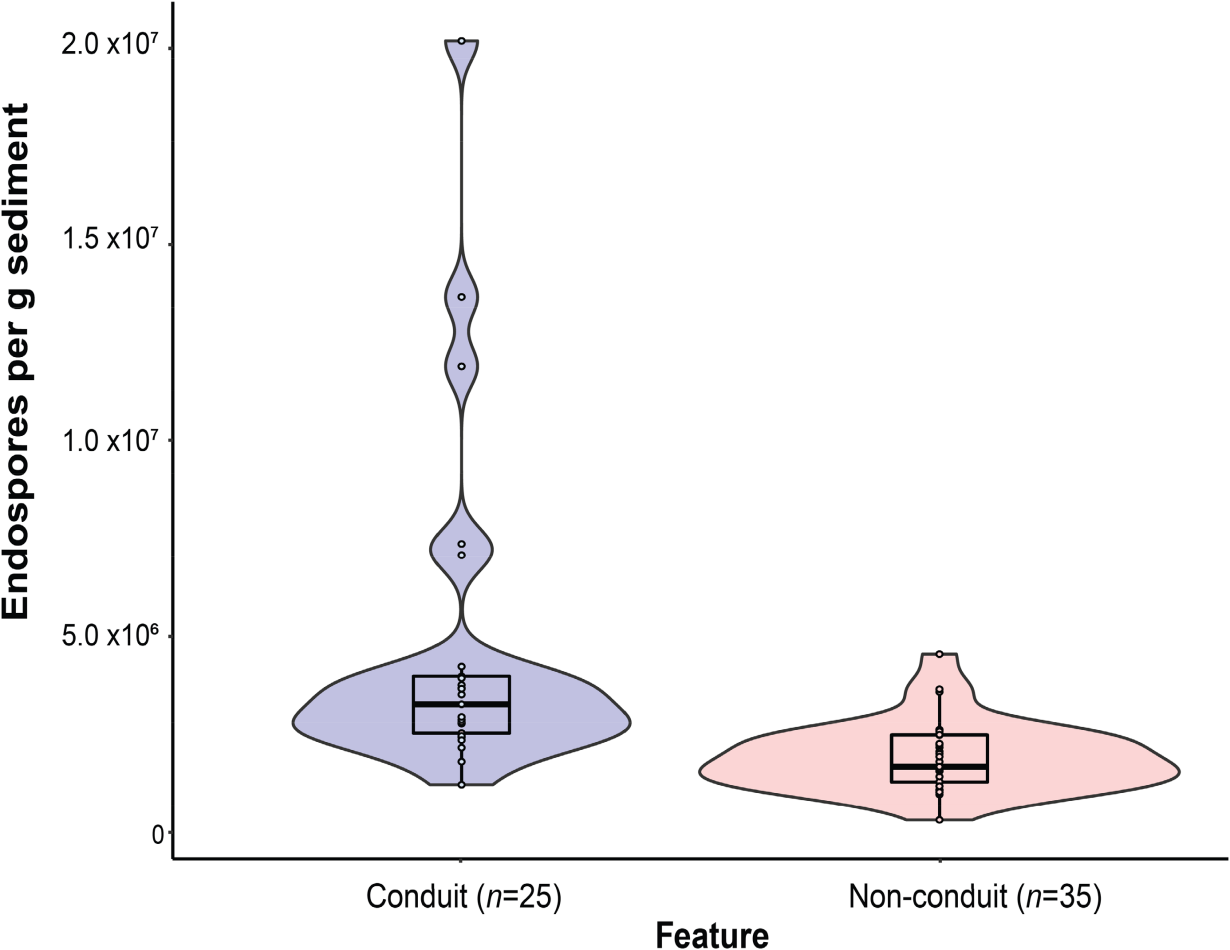
Violin plots show the distribution of endospore abundance in sediments in the presence or absence of subsurface conduits. The central thick black line represents the median and the interquartile range is defined by the surrounding black box. Significantly higher endospore abundances are observed at conduit sites (Wilcoxon rank sum/ Mann–Whitney *W* = 119, *p* < .01) in comparison to sediments lacking conduit features. There are no significant differences in the variance of endospore abundance in conduit vs non‐conduit locations (Bartlett K‐squared test *T* = 61.3, *p* < .01).

**FIGURE 4:**
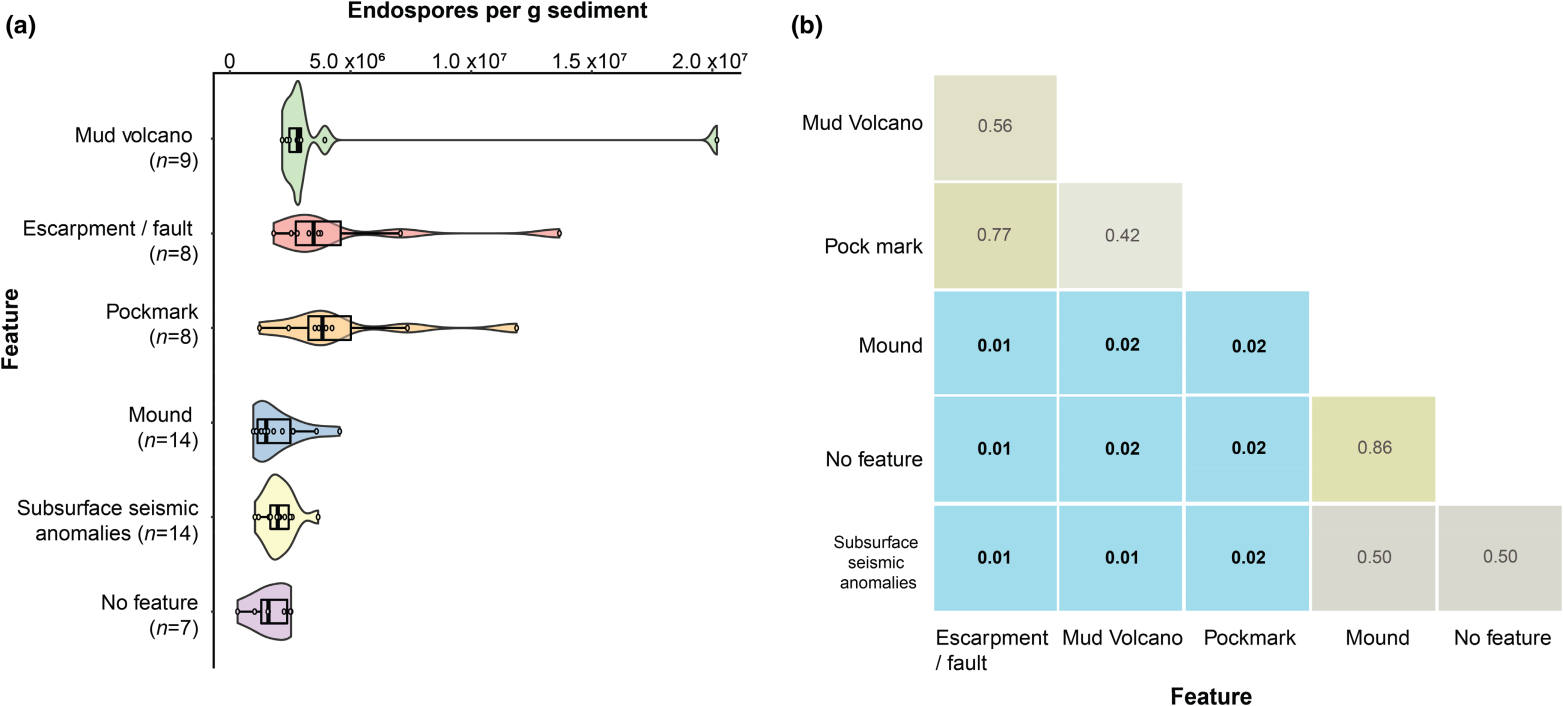
(a) Endospore abundance in 60 surface sediments grouped by marine geological seafloor features, as defined using compressed high intensity radiated pulse (CHIRP) sonar data. Violin plots show the spread of endospore abundance data, with the central thick black line representing the median and the surrounding black box defining the interquartile range. The number of sites with each feature type is shown in parentheses. (b) Pairwise comparison matrix of p‐values derived from endospore abundance classified by geological feature (a, *n* = 60). Heatmap contrasts p‐values for the Wilcoxon rank sum exact test adjusted using the method of Benjamini and Hochberg (1995). Significant differences between sample medians are shown in blue squares with bold text (*p* < .05), indicating there is a significant difference between conduit and non‐conduit groups but not within the two groups. Beige squares relate to sample medians with (*p* > .05) indicating the two sample medians come from the same population.

Surface sediment endospore levels ranged between 9.7 × 10^5^ and 3.0 × 10^7^ endospores per g sediment (Figure 1). The highest endospore abundances were found at conduit sites (Figure 3). Conduit sites showed considerable variability in endospore abundance (Figure 3), which was significantly higher than non‐conduit locations (Wilcoxon rank sum/Mann–Whitney *W* = 119, *p* < .01). Further assessing endospore abundance for each of six different feature types (Figure 4a) shows the highest median and variance at mud volcanoes, pockmarks and escarpment/faults. Conversely, the lowest endospore values and lowest variance are observed at mounds, subsurface seismic anomaly and no feature sites, with the lowest abundance (3.2 × 10^5^ endospores) found at no feature site EGM206. These relationships are summarised as pairwise comparisons between conduit and non‐conduit feature in Figure 4b.

**FIGURE 5:**
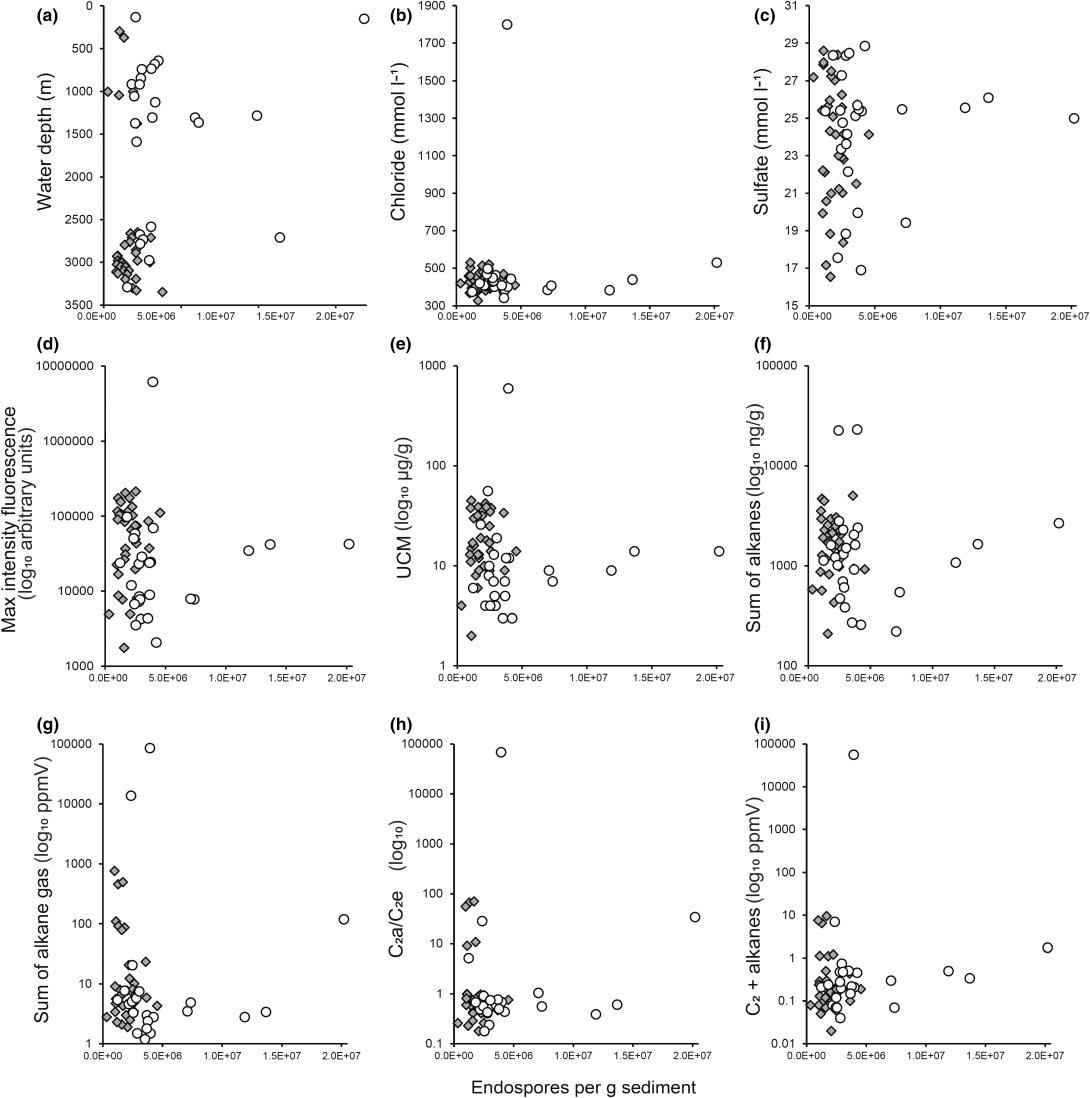
Surface sediment endospore abundance from 60 stations plotted against various physical/chemical variables, grey shaded diamonds show non‐conduit sites and white circles conduit sites. Panel (a) shows the majority of conduit sites are located in shallow waters. (b) Chloride concentrations show a slight trend of higher endospore abundance with higher chloride concentrations, but this is skewed by the presence of hypersaline conduit site EGM 116. (c) The highest endospore abundances occur together with sediment sulfate concentrations between 25 and 27 mmol l^−1^. In panels (d)‐(i) endospore abundance is plotted with log_10_ transformed geochemistry data (d) max intensity fluorescence (arbitrary units), (e) UCM; unresolved complex mixture (μg/g dry sediment), (f) sum of C_15_‐C_34_ n‐alkanes (ng/g dry sediment), (g) sum of total alkane gases (ppmV wet sediment), (h) C_2_a/C_2_e; the ratio of ethane to ethene (in ppmV concentrations), (i) C_2+_ alkanes; non‐methane (C_2+_) alkane gases.

The majority of conduits and the highest endospore abundances are found in shallower waters (Figure 5a; Table 1). Stations with significantly higher endospore abundances are, in decreasing order, EGM106 (mud volcano), 202 (escarpment/fault), 109 (pockmark), 111 (pockmark) and 117 (escarpment/fault). Analysis of sediment porewater geochemistry shows that sites with higher endospore abundances have no significant relationship to chloride concentrations (Figure 5b; Table 1) but are associated with higher sulphate concentrations (Figure 5c). The five stations mentioned above contribute to a trend of higher oil and gas geochemical indicators with higher endospore abundance (Figure 5d–i), but the majority of the data points show no direct correlation between total endospore abundance and biomarkers for migrated hydrocarbons (see Table S1 for additional analysis of geochemistry data).

**TABLE 1:** Pairwise comparison of endospore abundance classified by physical or chemical sediment variables (*n* = 60) analysed using the Wilcoxon rank sum (Mann–Whitney‐*U*) test (significant differences = *p* < .05)

Test variable	Cut‐off	Wilcoxon rank sum *p*‐value (test statistic)	Bartlett variance *p*‐value (test statistic)^a^
Shallow and deep vs. ultra‐deep depths	1500 m	<.01 (616)	<.01 (60.2)
Low vs. high chloride	19.5 g/L	.42 (393)	.07 (3.2)
Low vs. high sulphate	35 mm	.87 (169)	.02 (5.2)
Oil positive vs. negative	TSF < 50,000	.05 (348)	.56 (0.34)
Oil positive vs. negative	UCM of <10 μg/g sediment	.05 (348)	.56 (0.34)
Gas positive vs. negative	Measured using GC	.37 (225)	<.01 (33.9)

Abbreviations: TSF, total scanning fluorescence; UCM, unresolved complex mixture.

^a^ Testing using the Bartlett K‐squared test shows significant differences in the variance of two populations.

Heavy metals are prevalent in crude oils can be used as proxies to understand the origins and processes of oil formation (dos Anjos et al., 2018; Maryutina & Timerbaev, 2017; Mdluli et al., 2020; Sokol et al., 2018; Walkner et al., 2017). In marine sediments, heavy metals tend to be present in high concentrations in deep‐to‐shallow conduit features like faults, trenches and subduction zones, due to mineral dissolution with upward fluid flow (Cangemi et al., 2010; Welty et al., 2018). During subsurface fluid advection heavy metals are dissolved from surrounding minerals under high temperatures and pressures at depth, such that elevated concentrations in porewater can be used as a direct indication of fluid flow. Metals in the directly exchangeable porewater were chemically separated, and the data were sorted into conduit metal or non‐conduit metal groups for 27 out of the 60 sediment samples (Table S1). The general trend shows that higher endospore abundances are associated with higher metal concentrations, with mean porewater heavy metal concentrations found to be significantly higher at the conduit sites (Figure S1).

### 3.3 Distributions of thermospores in conduits and non‐conduit sites

**FIGURE 6:**
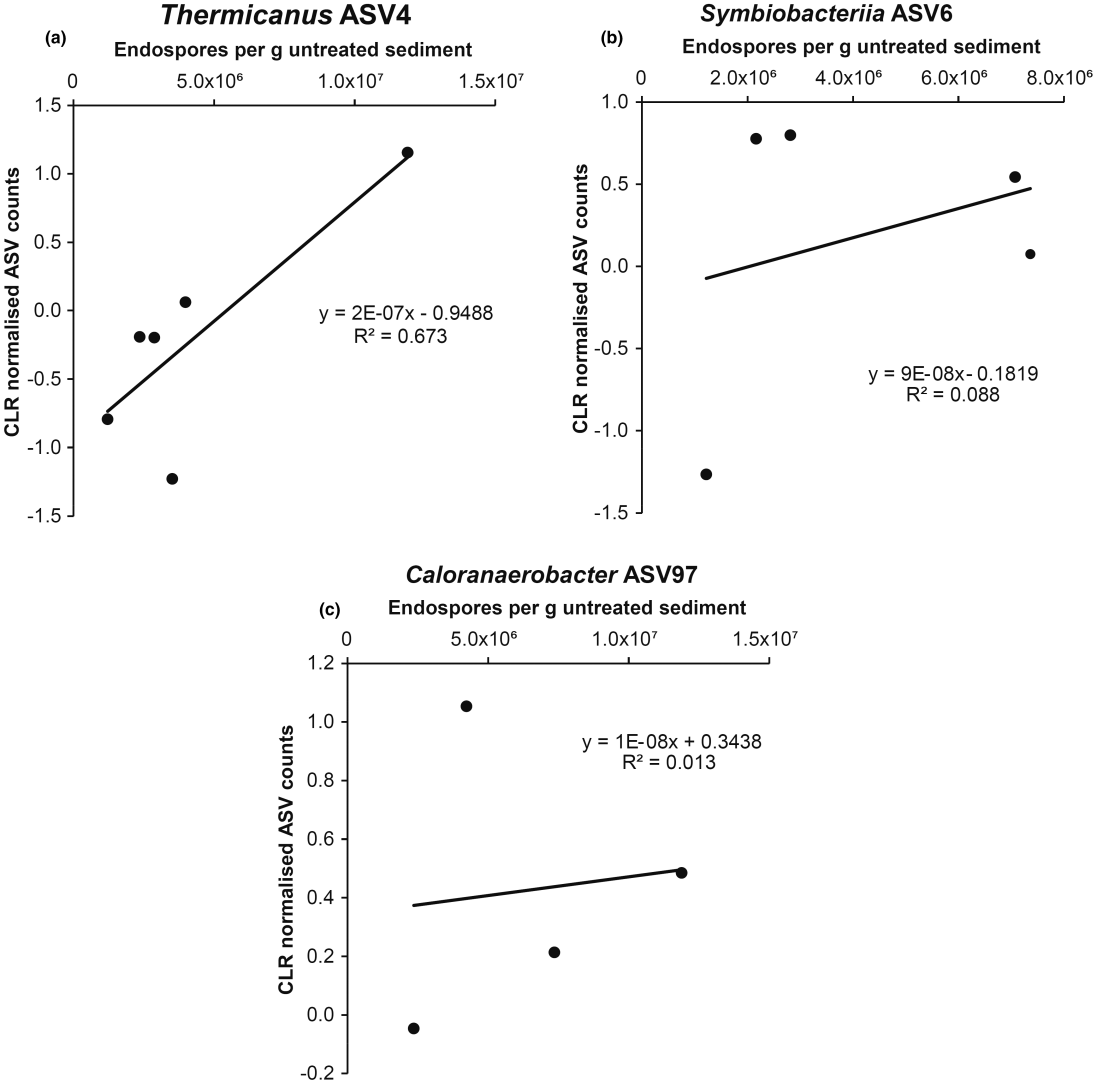
ASV normalised raw reads corresponding to germinated spores in conduit heated sediment after 14 days of incubation at 50°C. Scatterplots show centralised log ratio (CLR) normalised thermospore ASV relative abundance plotted against total endospore abundance as determined by DPA measurements, with correlation proportional to the number of stations the ASV was detected at. Additional ASVs unique to conduit sites but detected at too few sites to plot graphically (≤3 stations) are listed in Table 2.

Amplicon sequence variants belonging to the phylum *Firmicutes* increased in mean relative abundance from 3% to 91% following incubation at 50°C for 14 days. After heating, a total of 50 *Firmicutes* ASVs were identified in the conduit sediments, compared with 95 *Firmicutes* ASVs in non‐conduit sediments. Prior to incubation, non‐metric multidimensional scaling (NMDS) analysis of pairwise dissimilarities in *Firmicutes* ASV profiles revealed distinct clustering of the conduit and non‐conduit sites (Figure S2a), whereas after heating, less tight clustering and less distinct groupings were observed (Figure S2b). In heated sediments, higher relative abundances of *Bacilli*, *Moorellia* and *Symbiobacteria* are observed in conduit samples (Figure S2c) in comparison with higher relative abundances of *Desulfotomaculia*, *Sulfobacillia*, *Clostridia* (*Gelria*) and *Limnochordia* in non‐conduit samples (Figure S2d). Key ASVs identified as putative thermophiles that were exclusive to the conduit samples after heating are summarised in Table 2. These ASVs represent putative endospore‐forming thermophiles that contributed to the measured DPA concentration used to calculate the in situ endospore levels (Figures 1, 3, 4 and 5). Figure 6 highlights a selection of ASVs exclusive to conduit sediments following 14 days of sediment heating and detected in enough locations (>3) to assess the trend between overall endospore abundance and ASV relative abundance. *Thermicanus* (ASV4), *Symbiobacteriia* (ASV6) and *Caloranaerobacter* (ASV97) had higher 14‐day relative abundance at sites with higher in situ DPA concentrations. Table 2 shows that the closest relatives to ASVs 4, 6 and 97 are described as either thermophilic or originating from the deep biosphere. Table 2 further lists ASVs unique to the conduit sites but that were detected in too few locations to allow trend analyses (<3 stations) as for the three groups shown in Figure 6. This analysis does not reveal whether certain ASVs would have higher or lower relative abundance after a different period of high temperature incubation but does confirm that these populations are present as endospores in the seabed at these locations.

**TABLE 2:** *Firmicutes* ASVs identified exclusively in conduit site heated sediment slurry incubations but not identified in either unheated or heated non‐conduit samples

Genus	ASV ID	Closest relative in the NCBI database (% nucleotide identity)^a^	Description of closest related sequence
*Bacillus*	ASV3713	Uncultured bacterium (99.8%)	Dark fermentation reactor using lignocellulosic residues using heat treated (90°C) inoculum (Chatellard et al., 2016)
*Caloranaerobacter*	ASV97 ASV177 ASV1546 ASV6896	Uncultured bacterium (99.8%)	Fermentative thermophilic bacteria in the water column and sediment of Aarhus Bay, Denmark (Volpi et al., 2017)
*Desulfofarcimen*	ASV22	*Desulfofarcimen intricatum* (94.9%)	Sulphate‐reducing thermophile isolated from freshwater lake sediment (Watanabe et al., 2013)
*Hydrogenispora*	ASV18409	Uncultured bacterium (94.03%)	Isolated from a high and moderate temperature biogas reactor (Hirota et al., 2019)
*Moorella*	ASV16700	*Moorella stamsii* (100%)	Anaerobic thermophilic hydrogenogenic carboxydotroph isolated from digester sludge (Alves et al., 2013)
*Tepidanaerobacter*	ASV2528	Uncultured bacterium (99.8%)	Isolated from Ibeco Seal M‐90 bentonite buffer material at 50°C, used for spent nuclear fuel disposal (Pedersen et al., 2010)
*Tepidanaerobacter*	ASV18545	Uncultured bacterium (100%)	Anaerobic, syntrophic long‐chain fatty acid‐degrading microbe isolated from thermophilic methanogenic sludge (Hatamoto et al., 2007)
*Thermicanus*	ASV4	Uncultured *Thermicanus* sp. clone 2F2_PF 16S ribosomal RNA gene (100%)	Isolated from an oil production well at the Pampo Platform, Campos Basin, Brazil (Dellagnezze et al., 2016).
*Thermincola*	ASV890	Uncultured bacterium (97.0%)	Deep aquifer associated with accretionary prism, Ita‐wari well, Shimada, Shizuoka Prefecture, Japan (Kimura et al., 2010)
Unclassified	ASV6	*Symbiobacterium terraclitae* (93.7%)	Isolate in commensal relationship with *Bacillus*, typically thermophiles with high endospore production (Shiratori‐Takano et al., 2014).
Unclassified	ASV4546	Uncultured bacterium (99.5%)	Shimokita offshore drilling core sample, site C9001 at 1180.5 m water depth (Takami, H. & Arai, W., 2014, unpublished)

^a^ The percentage nucleotide sequence identity was determined using NCBI nucleotide blast search. *Firmicutes* ASVs listed in Table 2 were identified from conduit heated incubations but not observed in conduit site sediment pre‐incubation. Table 2 ASVs were additionally not identified in the non‐conduit samples, either prior to or after heated incubation.

## 4 DISCUSSION

### 4.1 Distribution of endospores in the EGM in relation to geology and sediment geochemistry

Salt tectonics are the largest active geological structures in the EGM region and relate to deformation of the Middle Jurassic Louann Salt which controls not only the types and location of tectonic salt structures but also sedimentation (e.g. via gravity spreading of salt tongues and sheets within sediments) and hydrocarbon accretion (Rowan et al., 1999; Wu et al., 1990). The evolution of the EGM region and the salt formations over the last 163–174 million years have resulted in highly complex geomorphological structures underlying the region. The lack of association between high endospore abundances and hypersalinity suggests there is no direct association with salt diapirism and that other factors have greater influence on endospore distribution. Typically, geochemical biomarker analyses are used to understand and locate hydrocarbons and fluid release. In this study, higher endospore abundances are observed at conduit sites but show no clear trends with geochemical biomarkers, except for the gas proxies C_2_a/C_2_e (the ratio of ethane to ethene) and non‐methane (C_2+_) alkane gases. These associations could be caused by as yet unknown effects of liquid hydrocarbons on endospore transport. It is also possible that the larger areas covered by the CHIRP results used to characterise sites as conduit or non‐conduit compared with the 10 cm diameter piston cores used for geochemistry and microbiology may highlight different sensitivities of the different approaches, potentially resulting in CHIRP‐based conduit classification without a strong signal from the piston cores. Additionally, conduit features may present intermittent gas or liquid hydrocarbon release due to complex pressure fluxes deep in the underlying geological system (Hovland & Judd, 1988; King & Maclean, 1970; Niemann & Boetius, 2010).

Dipicolinic acid is a non‐specific biomarker for bacterial endospores that does not discriminate between the large diversity of spore‐forming bacteria within the *Firmicutes* phylum. Endospores in large geographic basins such as the EGM likely originate from a variety of sources that remain connected to the seabed by different dispersal vectors. Accordingly, these spores and their vegetative counterparts also represent a variety of physiologies and conditions for promoting their germination. Spore‐forming *Firmicutes* are known to be abundant in wastewater and estuarine sediments (Atashgahi et al., 2015; Bell et al., 2018, 2020), shallow and deep‐sea marine sediments (Batzke et al., 2007; Fichtel, Koster, Rullkotter, et al., 2007; Fichtel et al., 2012; Hubert et al., 2012; Sass et al., 1997; Wörmer et al., 2019) and potentially from sub‐seabed high‐temperature sources (Chakraborty et al., 2018, 2020). Despite the Mississippi river suspended sediment discharge totalling around 145 million metric tons per year (Meade & Moody, 2010), endospore distribution in the Gulf of Mexico appears not to be directly influenced by terrestrial sources (Figure 1). If large numbers of endospores were discharged from the Mississippi river, this should be reflected in high‐DPA concentrations at locations proximal to the mouth of the river, that is stations EGM110, 112, 113 and 118, but this pattern is not observed. The association of heavy metal concentrations with endospores supports the theory that endospores are associated with liquid hydrocarbon discharge, probably as a mode of dispersal via the vertical migration of fluids. Moreover, the lack of association of endospores with iron concentrations in the sediment organic fraction (Figure S1b), and the known high‐Fe input from Mississippi river discharge (Presley et al., 1980) further supports the idea of endospore deep biosphere origins. These observations suggest other factors determine endospore distribution throughout the Gulf of Mexico.

Other deep biosphere studies using the DPA biomarker, including the work of Heuer et al. (2020) and Wörmer et al. (2019), have reported DPA concentrations that correspond to the low range of what are observed here in EGM non‐conduit sites. Figures 1 and 3 show that endospores in surface sediments are most abundant where the seabed is directly influenced by geological features, especially those associated with fluid flow conduits. Variability in the endospore abundance in surface sediments at conduit sites may also be related to whether the geological feature is currently actively transmitting subsurface fluids or not.

### 4.2 Dormant thermophile populations associated with EGM geological features

Sediment heating experiments resulted in *Firmicutes*‐dominated populations featuring putative sulphate‐reducing and sulphur‐oxidising bacteria from the orders *Desulfotomaculales* and *Sulfobacillales*, respectively. In relation to better understanding the potential of DPA as a biomarker, and the dispersal of endospores in marine environments, it is important to identify genera of *Firmicutes* found in conduit sediments where DPA concentrations are elevated. ASVs identified in heated sediment incubations revealed different populations of initially dormant endospores contributing to the measured DPA signals in different seabed locations. The most abundant ASVs in the heated sediment incubations were closely related (>99% sequence identity) to putative thermophilic bacteria, and in many cases to close relatives that have been detected in warm subsurface marine sediments.

These bacteria presumably transform into endospores when environmental conditions become unfavourable, potentially during, or prior to, geofluids transporting them away from warm anoxic habitats. Whereas the majority of the ASVs shared >99% sequence identity to bacteria in the Genbank database, four of the 14 ASVs exclusive to conduit sites (ASV6, ASV22, ASV890 and ASV18409; Table 2) were more distantly related, indicating potentially novel strains with fewer clues about their biogeography and dispersal histories. This might reflect dispersal from deep biosphere settings that have not been well characterised in previous studies, and that novel thermophiles may inhabit. ASVs that were detected in multiple locations were assessed in comparison with in situ endospore numbers estimated by DPA measurement in those sediments (Figure 6). For *Thermicanus* (ASV4), *Symbiobacteriia* (ASV6) and *Caloranaerobacter* (ASV97), higher endospore abundances correspond directly to greater enrichment after 14 days at 50°C. Endospores of thermophilic *Thermicanus*, *Symbiobacteriia* and *Caloranaerobacter* may therefore be contributing to the high‐DPA concentrations at conduit sites. Other factors may also influence these results, for example thermophilic endospores can contain significantly more DPA biomarker per endospore than mesospores or psychrospores (Fichtel, Koster, Rullkotter, et al., 2007; Rattray et al., 2021). Applying a widely used spore‐specific DPA conversion factor (Fichtel, Koster, Rullkotter, et al., 2007; Wörmer et al., 2019) may mask differences in endospore physiology between spore‐forming lineages and/or populations of spores in different sediment locations. For example, the high‐endospore abundances estimated at conduit sites may be inflated if thermospores with more DPA are contributing to the signal in these samples. Thermophiles having more DPA in their spores could be related to sporulation being initiated in environments with higher temperatures (Melly et al., 2002).

### 4.3 Are high endospore abundances driven by deep biosphere processes?

While the distribution of endospores in EGM sediments is complex, the data presented in this study suggest that geological features play a role in shaping the biogeography of spore‐forming bacteria in the marine environment. High endospore abundances at conduit sites (Figure 3) implies that these sites feature inputs of endospores originating from deeper sediment layers that support the metabolism of anaerobic thermophiles. Fluid venting from conduit systems such as mud volcanos, pockmarks and escarpments or faults occurs in response to fluid compression in the underlying rock that eventually depressurizes (Paull et al., 1984). Fluid release can span a range of intensities, from episodic to catastrophic (occurring in both pockmarks and mud volcanoes) to continual low‐level discharge (e.g. faults). Higher endospore abundances were measured at conduit sites that had relatively high sulphate and chloride concentrations; however, some sediments without any conduit features and exhibiting lower endospore abundances also have high levels of sulphate and chloride. Altogether this suggests that there are complex geological factors influencing the distribution of endospores, and that geofluid‐driven migration is not necessarily universally observed. Many questions still remain about the origins of endospores in the deep biosphere. Wörmer et al. (2019) showed that endospore abundance in global deep marine sediments increase with depth, presumably creating a seed bank waiting to re‐emerge. Further studies are needed on endospore abundance in relation to depth below the seafloor to properly understand the distribution of endospores in relation to geological structures in order to determine, among other things, whether these endospores are derived from the ancient genetic seed bank or are products of more recent biosynthesis.

## 5 CONCLUSIONS

Dipicolinic acid was measured to estimate endospore abundance in surface sediment samples from 60 locations representing various geological settings within the eastern Gulf of Mexico. Higher endospore abundances were observed at sites associated with CHIRP identified geofluid conduits including mud volcanoes, pockmarks, escarpments and faults that can potentially transport endospores from the deep biosphere up to the surface. Correlating endospore abundances with ASVs of thermophiles enriched in heated sediment incubations revealed that closely related bacteria are detected in warm environments, including deep biosphere and/or petroleum systems. These findings support that thermospores are biosynthesised in geologically generated hot subsurface environments. High abundances of dormant, recalcitrant endospores at geological seep features in the seabed point to the potential use of the dipicolinic acid biomarker for locating geofluid conduits to help better understand advective fluid flow in the EGM and other ocean basins.

## CONFLICT OF INTEREST

The authors declare they have no competing interests.

## Supporting information


Figures S1 and S2

Table S1

## Data Availability

The data that supports the findings of this study are available in the supplementary material of this article.

## References

[gbi12517-bib-0001] Aitchison, J. (1982). The statistical analysis of compositional data. Journal of the Royal Statistical Society. Series B, Statistical Methodology, 44(2), 139–160.

[gbi12517-bib-0002] Alves, J. I. , van Gelder, A. H. , Alves, M. M. , Sousa, D. Z. , & Plugge, C. M. (2013). *Moorella stamsii* sp. nov., a new anaerobic thermophilic hydrogenogenic carboxydotroph isolated from digester sludge. International Journal of Systematic and Evolutionary Microbiology, 63(Pt 11), 4072–4076. 10.1099/ijs.0.050369-0 23749275

[gbi12517-bib-0003] Atashgahi, S. , Aydin, R. , Dimitrov, M. R. , Sipkema, D. , Hamonts, K. , Lahti, L. , Maphosa, F. , Kruse, T. , Saccenti, E. , Springael, D. , Dejonghe, W. , & Smidt, H. (2015). Impact of a wastewater treatment plant on microbial community composition and function in a hyporheic zone of a eutrophic river. Scientific Reports, 5, 17284. 10.1038/srep17284 26607034 PMC4660315

[gbi12517-bib-0004] Batzke, A. , Engelen, B. , Sass, H. , & Cypionka, H. (2007). Phylogenetic and physiological diversity of cultured deep‐biosphere bacteria from equatorial Pacific Ocean and Peru margin sediments. Geomicrobiology Journal, 24(3–4), 261–273. 10.1080/01490450701456453

[gbi12517-bib-0005] Bell, E. , Blake, L. I. , Sherry, A. , Head, I. M. , & Hubert, C. R. J. (2018). Distribution of thermophilic endospores in a temperate estuary indicate that dispersal history structures sediment microbial communities. Environmental Microbiology, 20(3), 1134–1147. 10.1111/1462-2920.14056 29393553 PMC6849807

[gbi12517-bib-0006] Bell, E. , Sherry, A. , Pilloni, G. , Suarez‐Suarez, A. , Cramm, M. A. , Cueto, G. , Head, I. M. , & Hubert, C. R. J. (2020). Sediment cooling triggers germination and sulfate reduction by heat‐resistant thermophilic spore‐forming bacteria. Environmental Microbiology, 22(1), 456–465. 10.1111/1462-2920.14866 31742859

[gbi12517-bib-0007] Benitez, L. N. , & Dubois, J.‐P. (1999). Evaluation of the selectivity of sequential extraction procedures applied to the speciation of cadmium in soils. International Journal of Environmental Analytical Chemistry, 74(1–4), 289–303. 10.1080/03067319908031433

[gbi12517-bib-0008] Benjamini, Y. , & Hochberg, Y. (1995). Controlling the false discovery rate: A practical and powerful approach to multiple testing. Journal of the Royal Statistical Society. Series B, Statistical Methodology, 57(1), 289–300.

[gbi12517-bib-0009] Callahan, B. J. , McMurdie, P. J. , Rosen, M. J. , Han, A. W. , Johnson, A. J. A. , & Holmes, S. P. (2016). DADA2: High‐resolution sample inference from Illumina amplicon data. Nature Methods, 13(7), 581–583. 10.1038/nmeth.3869 27214047 PMC4927377

[gbi12517-bib-0010] Cangemi, M. , Di Leonardo, R. , Bellanca, A. , Cundy, A. , Neri, R. , & Angelone, M. (2010). Geochemistry and mineralogy of sediments and authigenic carbonates from the Malta plateau, strait of Sicily (Central Mediterranean): Relationships with mud/fluid release from a mud volcano system. Chemical Geology, 276(3–4), 294–308. 10.1016/j.chemgeo.2010.06.014

[gbi12517-bib-0011] Chakraborty, A. , Ellefson, E. , Li, C. , Gittins, D. , Brooks, J. , Bernard, B. B. , & Hubert, C. R. J. (2018). Thermophilic endospores associated with migrated thermogenic hydrocarbons in deep Gulf of Mexico marine sediments. The ISME Journal, 12(8), 1895–1906. 10.1038/s41396-018-0108-y 29599524 PMC6052102

[gbi12517-bib-0012] Chakraborty, A. , Ruff, S. E. , Dong, X. , Ellefson, E. D. , Li, C. , Brooks, J. M. , McBee, J. , Bernard, B. B. , & Hubert, C. R. J. (2020). Hydrocarbon seepage in the deep seabed links subsurface and seafloor biospheres. Proceedings of the National Academy of Sciences of the United States of America, 117(20), 11029–11037. 10.1073/pnas.2002289117 32354993 PMC7245137

[gbi12517-bib-0013] Chatellard, L. , Trably, E. , & Carrère, H. (2016). The type of carbohydrates specifically selects microbial community structures and fermentation patterns. Bioresource Technology, 221, 541–549. 10.1016/j.biortech.2016.09.084 27686722

[gbi12517-bib-0014] Dellagnezze, B. M. , Vasconcellos, S. P. D. , Melo, I. S. D. , Santos Neto, E. V. D. , & Oliveira, V. M. D. (2016). Evaluation of bacterial diversity recovered from petroleum samples using different physical matrices. Brazilian Journal of Microbiology, 47(3), 712–723. 10.1016/j.bjm.2016.04.004 27282730 PMC4927652

[gbi12517-bib-0015] dos Anjos, S. L. , Alves, J. C. , Soares, S. A. R. , Araujo, R. G. O. , de Oliveira, O. M. C. , Queiroz, A. F. S. , & Ferreira, S. L. C. (2018). Multivariate optimization of a procedure employing microwave‐assisted digestion for the determination of nickel and vanadium in crude oil by ICP OES. Talanta, 178, 842–846. 10.1016/j.talanta.2017.10.010 29136903

[gbi12517-bib-0016] Fichtel, J. , Koster, J. , Rullkotter, J. , & Sass, H. (2007). Spore dipicolinic acid contents used for estimating the number of endospores in sediments. FEMS Microbiology Ecology, 61(3), 522–532. 10.1111/j.1574-6941.2007.00354.x 17623026

[gbi12517-bib-0017] Fichtel, J. , Koster, J. , Scholz‐Bottcher, B. , Sass, H. , & Rullkotter, J. (2007). A highly sensitive HPLC method for determination of nanomolar concentrations of dipicolinic acid, a characteristic constituent of bacterial endospores. Journal of Microbiological Methods, 70(2), 319–327. 10.1016/j.mimet.2007.05.008 17573136

[gbi12517-bib-0018] Fichtel, K. , Mathes, F. , Könneke, M. , Cypionka, H. , & Engelen, B. (2012). Isolation of sulfate‐reducing bacteria from sediments above the deep‐subseafloor aquifer. Frontiers in Microbiology, 3, 65. 10.3389/fmicb.2012.00065 22363336 PMC3282481

[gbi12517-bib-0019] Forster, S. C. , Kumar, N. , Anonye, B. O. , Almeida, A. , Viciani, E. , Stares, M. D. , Dunn, M. , Mkandawire, T. T. , Zhu, A. , Shao, Y. , Pike, L. J. , Louie, T. , Browne, H. P. , Mitchell, A. L. , Neville, B. A. , Finn, R. D. , & Lawley, T. D. (2019). A human gut bacterial genome and culture collection for improved metagenomic analyses. Nature Biotechnology, 37(2), 186–192. 10.1038/s41587-018-0009-7 PMC6785715 30718869

[gbi12517-bib-0020] Garrity, C. P. , & Soller, D. (2009). *Database of the geologic map of North America— Adapted from the map by J.C. Reed, Jr. and others (2005)* (424) http://pubs.er.usgs.gov/publication/ds424

[gbi12517-bib-0021] Gloor, G. B. , Macklaim, J. M. , Pawlowsky‐Glahn, V. , & Egozcue, J. J. (2017). Microbiome datasets are compositional: And this is not optional. Frontiers in Microbiology, 8, 2224. 10.3389/fmicb.2017.02224 29187837 PMC5695134

[gbi12517-bib-0022] Hatamoto, M. , Imachi, H. , Ohashi, A. , & Harada, H. (2007). Identification and cultivation of anaerobic, syntrophic long‐chain fatty acid‐degrading microbes from mesophilic and thermophilic methanogenic sludges. Applied and Environmental Microbiology, 73(4), 1332–1340. 10.1128/AEM.02053-06 17189450 PMC1828665

[gbi12517-bib-0023] Heuer, V. B. , Inagaki, F. , Morono, Y. , Kubo, Y. , Spivack, A. J. , Viehweger, B. , Treude, T. , Beulig, F. , Schubotz, F. , Tonai, S. , Bowden, S. A. , Cramm, M. , Henkel, S. , Hirose, T. , Homola, K. , Hoshino, T. , Ijiri, A. , Imachi, H. , Kamiya, N. , … Hinrichs, K.‐U. (2020). Temperature limits to deep subseafloor life in the Nankai trough subduction zone. Science, 370(6521), 1230–1234. 10.1126/science.abd7934 33273103

[gbi12517-bib-0024] Hirota, K. , Miura, C. , Motomura, N. , Matsuyama, H. , & Yumoto, I. (2019). Isolation and identification of bacteria from high‐temperature compost at temperatures exceeding 90C. African Journal of Microbiology Research, 13(7), 134–144.

[gbi12517-bib-0025] Hoshino, T. , Doi, H. , Uramoto, G.‐I. , Wörmer, L. , Adhikari, R. R. , Xiao, N. , Morono, Y. , D'Hondt, S. , Hinrichs, K.‐U. , & Inagaki, F. (2020). Global diversity of microbial communities in marine sediment. Proceedings of the National Academy of Sciences of the United States of America, 117(44), 27587–27597. 10.1073/pnas.1919139117 33077589 PMC7959581

[gbi12517-bib-0026] Hovland, M. , & Judd, A. G. (1988). Seabed pockmarks and seepages: Impact on geology, biology and the marine environment (Vol. 293). Graham & Trotman.

[gbi12517-bib-0027] Hubert, C. R. J. , Oldenburg, T. B. P. , Fustic, M. , Gray, N. D. , Larter, S. R. , Penn, K. , Rowan, A. K. , Seshadri, R. , Sherry, A. , Swainsbury, R. , Voordouw, G. , Voordouw, J. K. , & Head, I. M. (2012). Massive dominance of Epsilonproteobacteria in formation waters from a Canadian oil sands reservoir containing severely biodegraded oil. Environmental Microbiology, 14(2), 387–404. 10.1111/j.1462-2920.2011.02521.x 21824242 PMC3490369

[gbi12517-bib-0028] Kapili, B. J. , Barnett, S. E. , Buckley, D. H. , & Dekas, A. E. (2020). Evidence for phylogenetically and catabolically diverse active diazotrophs in deep‐sea sediment. The ISME Journal, 14(4), 971–983. 10.1038/s41396-019-0584-8 31907368 PMC7082343

[gbi12517-bib-0029] Kaul, A. , Mandal, S. , Davidov, O. , & Peddada, S. D. (2017). Analysis of microbiome data in the presence of excess zeros. Frontiers in Microbiology, 8, 2114. 10.3389/fmicb.2017.02114 29163406 PMC5682008

[gbi12517-bib-0030] Kimura, H. , Nashimoto, H. , Shimizu, M. , Hattori, S. , Yamada, K. , Koba, K. , Yoshida, N. , & Kato, K. (2010). Microbial methane production in deep aquifer associated with the accretionary prism in Southwest Japan. The ISME Journal, 4(4), 531–541. 10.1038/ismej.2009.132 19956275

[gbi12517-bib-0031] King, L. H. , & Maclean, B. (1970). Pockmarks on the Scotian shelf. Geological Society of America Bulletin, 81(10), 3141–3148.

[gbi12517-bib-0032] Klindworth, A. , Pruesse, E. , Schweer, T. , Peplies, J. , Quast, C. , Horn, M. , & Glöckner, F. O. (2012). Evaluation of general 16S ribosomal RNA gene PCR primers for classical and next‐generation sequencing‐based diversity studies. Nucleic Acids Research, 41(1), e1. 10.1093/nar/gks808 22933715 PMC3592464

[gbi12517-bib-0033] Lomstein, B. A. , & Jørgensen, B. B. (2012). Pre‐column liquid chromatographic determination of dipicolinic acid from bacterial endospores. Limnology and Oceanography: Methods, 10, 227–233. 10.4319/lom.2012.10.227

[gbi12517-bib-0034] Lomstein, B. A. , Langerhuus, A. T. , D'Hondt, S. , Jørgensen, B. B. , & Spivack, A. J. (2012). Endospore abundance, microbial growth and necromass turnover in deep sub‐seafloor sediment. Nature, 484(7392), 101–104. 10.1038/nature10905 22425999

[gbi12517-bib-0035] Lozupone, C. , Lladser, M. E. , Knights, D. , Stombaugh, J. , & Knight, R. (2011). UniFrac: An effective distance metric for microbial community comparison. The ISME Journal, 5(2), 169–172. 20827291 10.1038/ismej.2010.133 PMC3105689

[gbi12517-bib-0036] Mariat, D. , Firmesse, O. , Levenez, F. , Guimarăes, V. D. , Sokol, H. , Doré, J. , Corthier, G. , & Furet, J. P. (2009). The firmicutes/bacteroidetes ratio of the human microbiota changes with age. BMC Microbiology, 9(1), 123. 10.1186/1471-2180-9-123 19508720 PMC2702274

[gbi12517-bib-0037] Maryutina, T. A. , & Timerbaev, A. R. (2017). Metal speciation analysis of petroleum: Myth or reality? Analytica Chimica Acta, 991, 1–8. 10.1016/j.aca.2017.08.036 29031291

[gbi12517-bib-0038] McMurdie, P. J. , & Holmes, S. (2013). Phyloseq: An R package for reproducible interactive analysis and graphics of microbiome census data. PLoS One, 8(4), e61217. 10.1371/journal.pone.0061217 23630581 PMC3632530

[gbi12517-bib-0039] McMurdie, P. J. , & Holmes, S. (2014). Waste not, want not: Why rarefying microbiome data is inadmissible. PLoS Computational Biology, 10(4), e1003531. 10.1371/journal.pcbi.1003531 24699258 PMC3974642

[gbi12517-bib-0040] Mdluli, N. S. , Nomngongo, P. N. , & Mketo, N. (2020). A critical review on application of extraction methods prior to spectrometric determination of trace‐metals in oily matrices. Critical Reviews in Analytical Chemistry, 52, 1–18. 10.1080/10408347.2020.1781591 32619354

[gbi12517-bib-0041] Meade, R. H. , & Moody, J. A. (2010). Causes for the decline of suspended‐sediment discharge in the Mississippi River system, 1940‐2007. Hydrological Processes, 24(1), 35–49. 10.1002/hyp.7477

[gbi12517-bib-0042] Melly, E. , Genest, P. C. , Gilmore, M. E. , Little, S. , Popham, D. L. , Driks, A. , & Setlow, P. (2002). Analysis of the properties of spores of *Bacillus subtilis* prepared at different temperatures. Journal of Applied Microbiology, 92(6), 1105–1115. 10.1046/j.1365-2672.2002.01644.x 12010551

[gbi12517-bib-0043] Muller, A. L. , de Rezende, J. R. , Hubert, C. R. J. , Kjeldsen, K. U. , Lagkouvardos, I. , Berry, D. , Jorgensen, B. B. , & Loy, A. (2014). Endospores of thermophilic bacteria as tracers of microbial dispersal by ocean currents. The ISME Journal, 8(6), 1153–1165. 10.1038/ismej.2013.225 24351936 PMC4030223

[gbi12517-bib-0044] Nash, D. (2013). 5.10 Tectonic geomorphology of Normal fault scarps. In J. F. Shroder (Ed.), Treatise on geomorphology (pp. 234–249). Academic Press.

[gbi12517-bib-0045] Niemann, H. , & Boetius, A. (2010). Mud Volcanoes. In K. N. Timmis (Ed.), Handbook of hydrocarbon and lipid microbiology (pp. 205–214). Springer.

[gbi12517-bib-0046] Oksanen, J. , Blanchet, F. G. , Kindt, R. , Legendre, P. , Minchin, P. R. , O'Hara, R. B. , Simpson, G. L. , Solymos, P. , Stevens, M. H. H. , & Wagner, H. (2014). Vegan: Community ecology package. R package version 2.2‐0 . http://CRAN.Rproject.org/package=vegan

[gbi12517-bib-0047] Paull, C. K. , Hecker, B. , Commeau, R. , Freeman‐Lynde, R. P. , Neumann, C. , Corso, W. P. , Golubic, S. , Hook, J. E. , Sikes, E. , & Curray, J. (1984). Biological communities at the Florida escarpment resemble hydrothermal vent taxa. Science, 226(4677), 965–967. 10.1126/science.226.4677.965 17737352

[gbi12517-bib-0048] Pedersen, K. , Arlinger, J. , Edlund, J. , Eriksson, L. , Lydmark, S. , Johansson, J. , Jägevall, S. , & Rabe, L. (2010). Microbiology of Olkiluoto and ONKALO groundwater. Results and interpretations, 2008–2009. Posiva Oy.

[gbi12517-bib-0049] Presley, B. J. , Trefry, J. H. , & Shokes, R. F. (1980). Heavy metal inputs to Mississippi Delta sediments. Water, Air, and Soil Pollution, 13(4), 481–494. 10.1007/BF02191849

[gbi12517-bib-0050] Rattray, J. E. , Chakraborty, A. , Li, C. R. , Elizondo, G. , John, N. , Wong, M. C. , Radovic, J. R. , Oldenburg, T. B. P. , & Hubert, C. R. J. (2021). Sensitive quantification of dipicolinic acid from bacterial endospores in soils and sediments. Environmental Microbiology, 23, 1397–1406. 10.1111/1462-2920.15343 33264453 PMC8048543

[gbi12517-bib-0051] Reichardt, N. , Duncan, S. H. , Young, P. , Belenguer, A. , McWilliam Leitch, C. , Scott, K. P. , Flint, H. J. , & Louis, P. (2014). Phylogenetic distribution of three pathways for propionate production within the human gut microbiota. The ISME Journal, 8(6), 1323–1335. 10.1038/ismej.2014.14 24553467 PMC4030238

[gbi12517-bib-0052] Rowan, M. G. , Jackson, M. P. A. , & Trudgill, B. D. (1999). Salt‐related fault families and fault welds in the northern Gulf of Mexico. AAPG Bulletin, 83, 1454–1484.

[gbi12517-bib-0053] RStudio Team . (2020). RStudio: Integrated development for R. RStudio, PBC http://www.rstudio.com/

[gbi12517-bib-0054] Sass, H. , Cypionka, H. , & Babenzien, H. D. (1997). Vertical distribution of sulfate‐reducing bacteria at the oxic‐anoxic interface in sediments of the oligotrophic Lake Stechlin. FEMS Microbiology Ecology, 22(3), 245–255. 10.1111/j.1574-6941.1997.tb00377.x

[gbi12517-bib-0055] Setlow, B. , Atluri, S. , Kitchel, R. , Koziol‐Dube, K. , & Setlow, P. (2006). Role of Dipicolinic acid in resistance and stability of spores of *Bacillus subtilis* with or without DNA‐protective α/β‐type small acid‐soluble proteins. Journal of Bacteriology, 188(11), 3740–3747. 10.1128/JB.00212-06 16707666 PMC1482921

[gbi12517-bib-0056] Setlow, P. (2007). I will survive: DNA protection in bacterial spores. Trends in Microbiology, 15(4), 172–180. 10.1016/j.tim.2007.02.004 17336071

[gbi12517-bib-0057] Setlow, P. (2011). Resistance of bacterial spores (pp. 319–332). ASM Press.

[gbi12517-bib-0058] Shanmugam, G. (2021). Chapter 7 ‐ Triggering mechanisms of downslope processes. In G. Shanmugam (Ed.), Mass transport, gravity flows, and bottom currents (pp. 273–307). Elsevier.

[gbi12517-bib-0059] Shiratori‐Takano, H. , Akita, K. , Yamada, K. , Itoh, T. , Sugihara, T. , Beppu, T. , & Ueda, K. (2014). Description of *Symbiobacterium ostreiconchae* sp. nov., *Symbiobacterium turbinis* sp. nov. and *Symbiobacterium terraclitae* sp. nov., isolated from shellfish, emended description of the genus Symbiobacterium and proposal of Symbiobacteriaceae fam. Nov. International Journal of Systematic and Evolutionary Microbiology, 64(Pt 10), 3375–3383. 10.1099/ijs.0.063750-0 25013225

[gbi12517-bib-0060] Sokol, E. , Kokh, S. , Kozmenko, O. , Novikova, S. , Khvorov, P. , Nigmatulina, E. , Belogub, E. , & Kirillov, M. (2018). Mineralogy and geochemistry of mud volcanic ejecta: A new look at old issues (a case study from the Bulganak field, northern Black Sea). Minerals, 8(8), 344.

[gbi12517-bib-0061] Volpi, M. , Lomstein, B. A. , Sichert, A. , Røy, H. , Jørgensen, B. B. , & Kjeldsen, K. U. (2017). Identity, abundance, and reactivation kinetics of thermophilic fermentative endospores in cold marine sediment and seawater. Frontiers in Microbiology, 8, 131. 10.3389/fmicb.2017.00131 28220111 PMC5292427

[gbi12517-bib-0062] Walkner, C. , Gratzer, R. , Meisel, T. , & Bokhari, S. N. H. (2017). Multi‐element analysis of crude oils using ICP‐QQQ‐MS. Organic Geochemistry, 103, 22–30. 10.1016/j.orggeochem.2016.10.009

[gbi12517-bib-0063] Watanabe, M. , Kojima, H. , & Fukui, M. (2013). *Desulfotomaculum intricatum* sp. nov., a sulfate reducer isolated from freshwater lake sediment. International Journal of Systematic and Evolutionary Microbiology, 63(Pt 10), 3574–3578. 10.1099/ijs.0.051854-0 23584284

[gbi12517-bib-0064] Welty, C. J. , Sousa, M. L. , Dunnivant, F. M. , & Yancey, P. H. (2018). High‐density element concentrations in fish from subtidal to hadal zones of the Pacific Ocean. Heliyon, 4(10), e00840. 10.1016/j.heliyon.2018.e00840 30320235 PMC6180415

[gbi12517-bib-0065] Wickham, H. (2016). ggplot2: Elegant graphics for data analysis. Springer‐Verlag https://ggplot2.tidyverse.org

[gbi12517-bib-0066] Wong, R. G. , Wu, J. R. , & Gloor, G. B. (2016). Expanding the UniFrac toolbox. PLoS One, 11(9), e0161196. 27632205 10.1371/journal.pone.0161196 PMC5025018

[gbi12517-bib-0067] Wörmer, L. , Hoshino, T. , Bowles, M. W. , Viehweger, B. , Adhikari, R. R. , Xiao, N. , Uramoto, G.‐I. , Konneke, M. , Lazar, C. S. , Morono, Y. , Inagaki, F. , & Hinrichs, K.‐U. (2019). Microbial dormancy in the marine subsurface: Global endospore abundance and response to burial. Science Advances, 5(2), eaav1024. 10.1126/sciadv.aav1024 30801015 PMC6382399

[gbi12517-bib-0068] Wu, S. , Bally, A. W. , & Cramez, C. (1990). Allochthonous salt, structure and stratigraphy of the North‐Eastern Gulf of Mexico. part II: Structure. Marine and Petroleum Geology, 7(4), 334–370. 10.1016/0264-8172(90)90014-8

[gbi12517-bib-0069] Yang, W. , & Ponce, A. (2011). Validation of a clostridium endospore viability assay and analysis of Greenland ices and Atacama desert soils. Applied and Environmental Microbiology, 77(7), 2352–2358. 10.1128/AEM.01966-10 21296951 PMC3067429

[gbi12517-bib-0070] Zimmerman, A. J. , & Weindorf, D. C. (2010). Heavy metal and trace metal analysis in soil by sequential extraction: A review of procedures. International Journal of Analytical Chemistry, 2010, 387803. 10.1155/2010/387803 20414344 PMC2855982

