# Supplementary material for: Endospores associated with deep seabed geofluid features in the eastern Gulf of Mexico
Source: Geobiology. 2022 Aug 22;20(6):823–36. doi: 10.1111/gbi.12517 (PMC9804197; doi:10.1111/gbi.12517)
Supplement: Supplementary file 1 — Figures S1 and S2 [file GBI-20-823-s002.docx]

**Supplementary information**

**Table S1. Sample metadata see excel sheet S1**


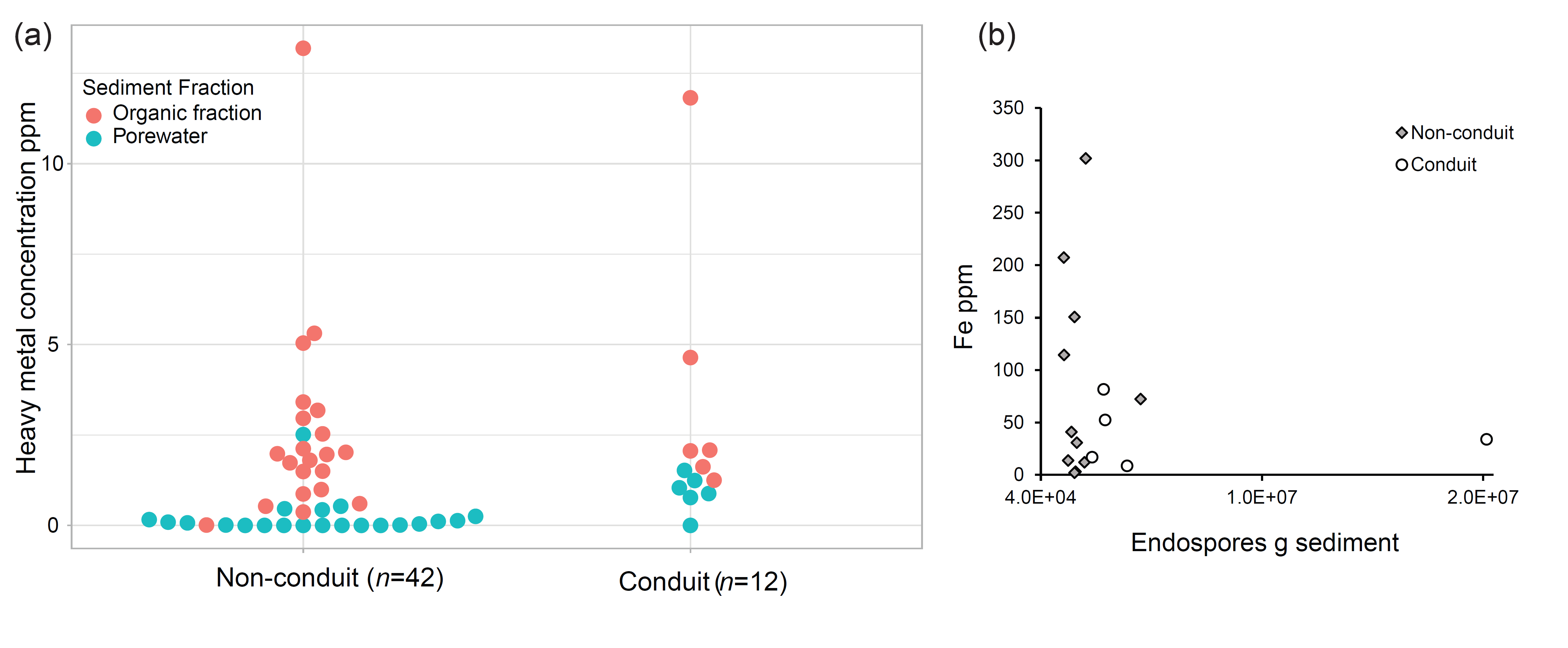


**Figure S1**. (a) Surface sediment heavy metal concentrations (summed heavy metals per sediment fraction per sample) plotted by the presence or absence of a conduit feature. Heavy metals included in the summed concentration are As, Co, Cr, Cu, Ga, Ni, Mo and Zn. Heavy metals were sequentially stripped from the sediment and the biologically available metals in the pore water and organic fractions are shown. Wilcoxon rank sum test of the data indicated the median heavy metal concentrations in the non-conduit samples are significantly different (p=<0.5) from the median heavy metal concentrations in the conduit samples. No significant difference was observed between heavy metals associated with the organic fraction in the conduit or no conduit populations. (b) Surface sediment organic fraction Fe concentrations (ppm) plotted with the abundance of endospores per g sediment. Fe concentrations are higher at non-conduit sites with low endospore abundance.


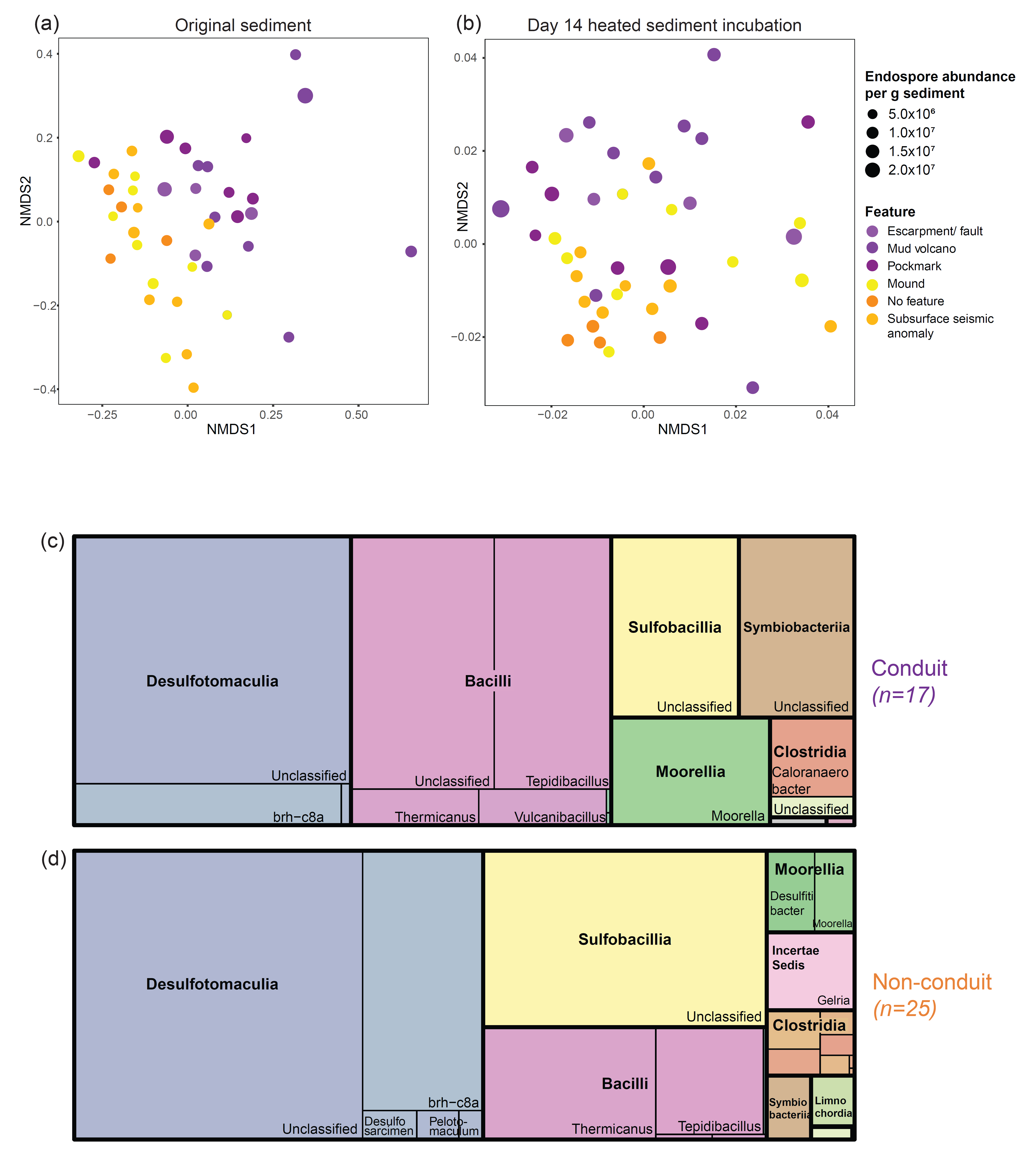


**Figure S2.** NMDS (non-metric multidimensional scaling) ordination plotting the two-dimensional configuration of bacterial ASVs in 42 sediment samples in (a) the original sediment samples and (b) the samples after 14 days of heated incubation at 50°C. ASV analysis is based on Bray-Curtis (dis)similarities from root-transformed abundances (stress = (a) 0.1 (b) 0.2, using Kruskal’s stress formula). Conduit sites are shown in purple and no-conduit sites in yellow/orange. The size of the circles corresponds to endospore abundance determined in the original sediment. Lower panels contain treemaps of the average composition of day 14 heated sediment samples in (c) conduit and (d) no-conduit sites. Several key species have been identified in the heated sediment that were under the limit of identification in the untreated sediment, see Table 2.
